# DNA Gyrase Is the Target for the Quinolone Drug Ciprofloxacin in Arabidopsis thaliana*

**DOI:** 10.1074/jbc.M115.689554

**Published:** 2015-12-09

**Authors:** Katherine M. Evans-Roberts, Lesley A. Mitchenall, Melisa K. Wall, Julie Leroux, Joshua S. Mylne, Anthony Maxwell

**Affiliations:** From the ‡ Department of Biological Chemistry, John Innes Centre, Norwich Research Park, Norwich NR4 7UH, United Kingdom,; the § School of Chemistry and Biochemistry, University of Western Australia, 35 Stirling Highway, Crawley, Perth 6009, Australia, and; the ¶ Australian Research Council Centre of Excellence in Plant Energy Biology, 35 Stirling Highway, Crawley, Perth 6009, Australia

**Keywords:** chloroplast, DNA gyrase, DNA replication, DNA topoisomerase, plant biochemistry, quinolones

## Abstract

The *Arabidopsis thaliana* genome contains four genes that were originally annotated as potentially encoding DNA gyrase: *ATGYRA*, *ATGYRB1*, *ATGYRB2*, and *ATGYRB3.* Although we subsequently showed that *ATGYRB3* does not encode a gyrase subunit, the other three genes potentially encode subunits of a plant gyrase. We also showed evidence for the existence of supercoiling activity in *A. thaliana* and that the plant is sensitive to quinolone and aminocoumarin antibiotics, compounds that target DNA gyrase in bacteria. However, it was not possible at that time to show whether the *A. thaliana* genes encoded an active gyrase enzyme, nor whether that enzyme is indeed the target for the quinolone and aminocoumarin antibiotics. Here we show that an *A. thaliana* mutant resistant to the quinolone drug ciprofloxacin has a point mutation in *ATGYRA*. Moreover we show that, as in bacteria, the quinolone-sensitive (wild-type) allele is dominant to the resistant gene. Further we have heterologously expressed *ATGYRA* and *ATGYRB2* in a baculovirus expression system and shown supercoiling activity of the partially purified enzyme. Expression/purification of the quinolone-resistant *A. thaliana* gyrase yields active enzyme that is resistant to ciprofloxacin. Taken together these experiments now show unequivocally that *A. thaliana* encodes an organelle-targeted DNA gyrase that is the target of the quinolone drug ciprofloxacin; this has important consequences for plant physiology and the development of herbicides.

## Introduction

DNA topoisomerases control the topology of DNA in all cellsand have key roles in DNA replication and transcription (1, 2). They are classified into two types, I and II, depending upon whether their reactions proceed via transient single (I)- or double (II)-stranded breaks in DNA. All type II enzymes are able to relax DNA, and DNA gyrase, originally discovered in bacteria (3), can also introduce negative supercoils in a reaction coupled to ATP binding/hydrolysis (4). Due to their essential nature, topoisomerases have become key targets for anticancer drugs and antibiotics (4, 5). For example, bacterial gyrase is the target of aminocoumarins (*e.g.* novobiocin) and quinolones (*e.g.* ciprofloxacin).

Although DNA gyrase is an archetypal bacterial enzyme and is absent from most eukaryotes, there is evidence for its existence in plants and apicomplexans (6–8). Plant gyrase is targeted to organelles (chloroplasts and mitochondria); given their prokaryotic origins, this provides a rationale for the presence of gyrase in plants (6, 7). However, the exact role of gyrase in plant physiology and its specific function in organelles remain to be established, although it is likely to have a role in supporting organellar replication. The presence and apparent essentiality of gyrase in plants present opportunities for herbicide targeting; indeed it has been shown that *Arabidopsis thaliana* is sensitive to both the quinolone and the aminocoumarin antibiotics, implying that a functional gyrase is essential for *A. thaliana* development (7).

Initially, four putative gyrase genes were found in the *A. thaliana* genome (*ATGYRA* (At3g10690), *ATGYRB1* (At3g10270), *ATGYRB2* (At5g04130), and *ATGYRB3* (At5g04110)), but subsequent work showed that *ATGYRB3* does not encode a gyrase subunit (9). In addition, yeast two-hybrid analysis showed interaction of AtGyrA with AtGyrB1 but not with AtGyrB2; the significance of this result is unclear (9).

Although it is clear that gyrase genes are present in *A. thaliana*, that gyrase is essential for *A. thaliana* development, and that DNA supercoiling activity can be found in chloroplast and mitochondrial extracts (7), it remains to be shown whether these genes are responsible for generating an active gyrase enzyme. Indeed previous attempts to express active *A. thaliana* gyrase in *Escherichia coli* were unsuccessful (7). The toxicity to *A. thaliana* of aminocoumarin and quinolones (known gyrase inhibitors) provides some indication that active gyrase is present in plants (7); however these compounds are known to have other targets (10, 11). Here to determine whether the gyrase genes in *A. thaliana* encode an active enzyme that is the target for ciprofloxacin, we use forward genetics and reveal that the genetic basis for ciprofloxacin resistance *in vivo* is an amino acid change in *ATGYRA*, which we confirm by genetic rescue. We went on to make recombinant *A. thaliana* gyrase and show the same mutation confers ciprofloxacin-resistant supercoiling activity *in vitro*. These results demonstrate that *A. thaliana* contains a functional gyrase that is the direct target of ciprofloxacin. Aside from the important consequences for our understanding of plant physiology, these findings potentiate the development of herbicides targeted to plant gyrase.

## Experimental Procedures

### 

#### 

##### RNAi Experiments

**FIGURE 1.**
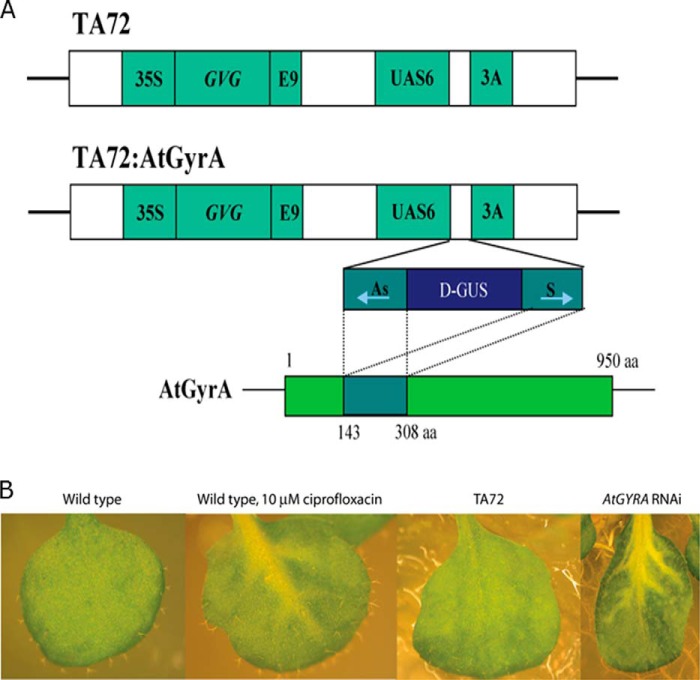
**RNAi knockdowns of *ATGYRA*.** *A*, plasmids pTA72 and pTA72:AtGyrA. The TA72 plasmid contains the chimeric transcription factor GVG between the cauliflower mosaic virus 35S promoter and the pea rbcS-E9 poly(A) sequence. It also contains six copies of the GAL4 upstream activation sequence and the pea rbcS-3A poly(A) sequence. The pTA72:AtGyrA plasmid contains a portion of the *ATGYRA* gene in both the sense and antisense orientations, separated by a 1,022-bp fragment of the GUS gene, and downstream of the GAL4 activation sequence. (Plasmids were donated by Hye Sun Cho.) *aa*, amino acids. *B*, leaves from 5-week-old *A. thaliana* plants. From *left* to *right*: wild type; wild type transferred to 10 μm ciprofloxacin after 4 weeks; a plant containing the RNAi control construct TA72 and transferred to 5 μm dexamethasone after 4 weeks; and a plant containing the ATGYRA RNAi construct and transferred to dexamethasone after 4 weeks.

To determine whether disabling *ATGYRA* results in the same phenotype as observed in the presence of ciprofloxacin (7), we utilized an inducible knock-out line of *A. thaliana* (gift of Hye Sun Cho, Korean Research Institute of Bioscience and Biotechnology), which employs the binary vector pTA72, based on the two-component glucocorticoid system (12). (Lines of *A. thaliana* containing T-DNA insertions in *ATGYRA* are available, but these are seedling-lethal (7).) This system uses a chimeric transcription factor consisting of the hormone-binding domain of the glucocorticoid receptor protein, the DNA-binding domain from the yeast transcription factor GAL4, and the transactivating domain from the herpes viral protein VP16. The DNA sequence of interest is introduced such that it is transcribed from a promoter containing six copies of the GAL4 upstream activating sequence. It is flanked at the 3′ end by the poly(A) addition sequence of the pea rbcS-3A gene (which encodes the small subunit of ribulose-bisphosphate carboxylase). Thus, when a glucocorticoid, such as dexamethasone, is introduced, the chimeric transcription factor is produced, which induces expression of the DNA of interest. A control line of *A. thaliana* containing the pTA72 plasmid, in which no gene was controlled by the GAL4 upstream activating sequence, was also used. The other line contained the pTA72:ATGYRA RNAi plasmid (see Fig. 1*A*). In this plasmid, the antisense and sense sequences encoding amino acids 143–308 of *ATGYRA* were separated by a 1,022-bp fragment of the *GUS* gene and inserted downstream of the GAL4 activating sequence. In the presence of a glucocorticoid, the *ATGYRA* RNAi construct should be expressed, resulting in a hairpin structure of double-stranded ATGYRA mRNA with a *GUS* linker. The simultaneous expression of sense and antisense RNA to create double-stranded RNA in this way has been shown to interfere with gene expression in plants (13).

Both the control and the *ATGYRA* RNAi seeds were sterilized, vernalized, and grown on GM^4^ plates containing 12.5 μg/ml hygromycin. 4 weeks later, the resistant plants were transferred to GM plates containing 12.5 μg/ml hygromycin and 5 μm dexamethasone to induce expression of the RNAi construct (GM = Murashige and Skoog (MS) salts (4.7 g/liter), glucose (10.0 g/liter), *myo*-inositol (100 mg/liter), thiamine (1 mg/ml), pyridoxine (0.5 mg/liter), nicotinic acid (0.5 mg/liter), MES (1.5 g/liter), adjusted to pH 5.7 using 10 m KOH; 9 g/liter Phytagar was added). Wild-type plants were grown on drug-free growth media for 4 weeks, and then half were transferred to media containing 10 μm ciprofloxacin. A week later, chlorosis could be seen in the veins of growing leaves of both the dexamethasone-induced RNAi plants and the wild-type plants that were transferred to ciprofloxacin (see Fig. 1*B*).

##### Mutagenesis

EMS-mutagenized *A. thaliana* M_2_ seeds were purchased from Lehle Seeds (catalogue number M2E-02-03). Approximately 400,000 M_2_ seeds were sterilized, stratified, and grown on GM plates containing 5 μm ciprofloxacin. 40 m_2_ plants were grown from different parental groups, with each parental group consisting of ∼1,192 ± 56 m_1_ plants. The offspring of ∼48,000 M_1_ plants were screened.

##### Electron Microscopy

To examine the effect of ciprofloxacin on organelles, wild-type and resistant plants were grown on GM agar plates for 4 weeks at 22 °C under a 16-h-long-day photoperiod and then transferred to plates containing 0, 1, or 10 μm ciprofloxacin for 2 weeks. Leaves were fixed with Ga/Os, embedded in resin, and cut into sections using an ultramicrotome. The sections were contrast-stained with uranyl citrate and lead citrate. They were then examined using a Jeol 1200 EX transmission electron microscope. Approximately 100 different fields of view were examined by transmission electron microscopy.

##### Rescue of Genomic gyra-3

To determine whether the A212V mutation in *gyra-3* causes ciprofloxacin resistance, we transformed *gyra-3* with a WT genomic copy of *ATGYRA*. A 7,755-bp genomic DNA fragment containing *ATGYRA* (At3g10690) was amplified by PCR using the bacterial artificial chromosome T7M13 as template, *Pfu* Ultra High Fidelity DNA polymerase (Agilent), and two primers (JM635 and JM637). JM635 (5′-acg atc gaT TTG TGA AGG TGC GGA GCC ACT GAT-3′) binds 802 bp upstream of the *ATGYRA* start ATG and adds a ClaI site (underlined) for subcloning and JM637 (5′-aaa gag ctc GAT TAT GGG GTT TGG TGC TTA CGT C-3′), which binds 100 bp downstream of the predicted *ATGYRA* 3′-UTR and adds a SacI site (underlined) for subcloning. (Primer sequence that does not match the genomic DNA is denoted by lowercase.) The *ATGYRA* fragment was A-tailed using *Taq* DNA polymerase before being cloned into pGEM-T Easy (Promega). The *ATGYRA* fragment was liberated from pGEM-*GYRA* by digestion with SacI and SalI. This SalI-GYRA-SacI fragment was ligated into the binary vector pSLJ755I5 (14) that had been linearized by digestion with XhoI and SacI (SalI and XhoI have compatible ends for ligation). The final pSLJ755I5-*GYRA* construct was triparentally mated into *Agrobacterium tumefaciens* strain LBA4404 and used to transform *gyra-3* by floral dipping (15). The first transgenic generation (T1) was selected with Basta® herbicide, and the segregation of Basta resistance in their progeny (T2) was used to establish the number of T-DNA loci. Three lines were selected for further study as they segregated for Basta resistance as a single T-DNA locus. Homozygous lines were identified using Basta resistance in the T3. To follow the *gyra-3* mutation, we developed a Derived Cleaved Amplified Polymorphic Sequences (dCAPS) assay using dCAPS Finder 2.0 (16). The primer pair JM633 (5′-TTG GCT CAA TAG ATG CAG ATC CTC CTG CaG-3′) and JM634 (5′-GCT GAA AAC ACT GAC AAA AAT ACA-3′) were used to amplify a 221-bp *ATGYRA* product (mismatch in JM633 is denoted by lowercase). The wild-type PCR product is cleaved into 191 and 30 bp by AluI digestion, whereas the *gyra-3* PCR product will not cleave with AluI. To assess the effect of the *ATGYRA* transgene on *gyra-3*, three transgenic lines homozygous for the *ATGYRA* transgene in *gyra-3* were sown with controls WT Col-0 and *gyra-3*. The seeds were sown on drug-free MS-agar plates (1% agar, 1× MS, pH 5.7, 1% glucose), plates containing 25 μm glufosinate ammonium (Sigma Aldrich, catalog number 45520), plates containing 5 μm ciprofloxacin (Sigma Aldrich, catalog number 17850), and finally plates containing both 25 μm glufosinate ammonium and 5 μm ciprofloxacin. Plates were stratified for 3 days at 4 °C and grown at 22 °C for 21 days under 16-h light/8-h dark conditions and then imaged.

##### Complementation of Temperature-sensitive Mutants

The AtGyrA(A212V) mutation was introduced into *ATGYRA* (lacking its transit peptide) by site-directed mutagenesis and cloned into pET17b (Novagen). Plasmids containing wild-type and mutant *ATGYRA* ORFs were transformed into the *E. coli* temperature-sensitive *gyrA* strain KNK453 (17) by electroporation. Ampicillin-resistant colonies were then streaked onto duplicated LB plates, which were incubated at 30 and 42 °C overnight. The *ATGYRA* genes were also cloned under the control of an *E. coli gyrB* promoter using vector pKER15 (9). An XhoI site was introduced at the 5′ end and a NotI site was introduced at the 3′ end of *ATGYRA* in pKER3 (18). As a control, *E. coli gyrA* was also ligated into the pKER15 plasmid; this gene was amplified by PCR from plasmid pPH3 (19) using the primers 5′-CTC GAG ATG AGC GAC CTT GC-3′ and 5′-GCG GCC GCT TAT TCT TCT TCT GGC-3′. The PCR product was gel-purified and ligated into pGEM-T Easy (Promega). An internal XhoI site in the *E. coli gyrA* clone was removed without affecting the protein sequence by site-directed mutagenesis. *ATGYRA* and *E. coli gyrA* were then subcloned into pKER15 to create plasmids pKER23 and pKER26, respectively. These plasmids were transformed into KNK453, and also into EJ45, another *gyrA^ts^* strain (20).

##### Heterologous Protein Expression

Previous multiple attempts to heterologously express the *ATGYRA* and *ATGYRB* genes in *E. coli*, *Nicotiana benthamiana*, and other expression systems were only partially successful (7, 9). Successful expression has been achieved using insect cells (baculovirus vectors) initiated by work in collaboration with the Oxford Protein Production Facility (OPPF; Harwell, UK). The wild-type *ATGYRA* and *ATGYRB2* genes, lacking the sequences encoding the putative transit peptides, were cloned separately into pOPINF (21) using the In-Fusion system (Clontech) and co-expressed in Sf9 insect cells at the OPPF. After initial trials and optimization by the OPPF, the expression was continued at the John Innes Centre. Sf21 cells (0.5 × 10^5^) were transfected with a mixture of linearized Bacmid 1629 (22), FuGENE HD (Promega), and the pOPINF constructs containing the *ATGYRA* and *ATGYRB2* genes in 2 ml of Sf9011 media (Invitrogen); pVSV_GTM_GFP, a transfer vector encoding GFP was used as a positive control (23). These were grown in 6-well plates without shaking for 7 days, after which the supernatant was removed from the cells and kept as P1 viral stock; this was used to infect 25 ml of Sf21 cells at 1 × 10^6^/ml in Sf9011 media to determine the level of infection and the incubation time for optimal co-expression. The cells were grown in shaking 75-cm^2^ tissue culture flasks. The supernatants from these trials were kept as P2 viral stock. Cells from a final large scale culture using 700 ml of Sf21 cells at 1 × 10^6^cells/ml infected with 5 ml of P2 viral stock in Sf9011 media were shaken at 27 °C in 2-liter conical flasks for 72 h. The cells were pelleted and then resuspended in TGEB100 and broken with a cell disrupter. The His_6_-tagged enzyme was partially purified with an imidazole gradient (0.02–1 m) on a nickel column (HiTrap FF, GE Healthcare), the peak fractions were dialyzed into TGEB100, and the pooled fractions were tested for activity. AtGyrA containing the A212V mutation was also co-expressed with AtGyrB2 as above.

##### DNA Gyrase Assays

Gyrase supercoiling assays (using relaxed DNA as a substrate) and quinolone-induced DNA cleavage assays were performed as described previously (24) except that the assay buffer was 50 mm HEPES·KOH (pH 7.9), 6 mm magnesium acetate, 4 mm DTT, 1 mm ATP, 100 mm potassium glutamate, 2 mm spermidine, 0.05 mg/ml albumin in a 30-μl reaction. Samples were incubated at 37 °C for 5 min, and the reaction was stopped with the addition of 20 μl of 40% sucrose, 100 mm Tris HCl (pH 8), 100 mm EDTA, 0.5 mg/ml bromphenol blue, and 30 μl of chloroform/isoamyl alcohol (24:1). Samples were analyzed on 1% w/v agarose gels in 40 mm Tris acetate (pH 8.0), 1 mm EDTA, at 80 V for 2 h. Gel images were captured using a Syngene documentation system and analyzed using ImageJ.

## Results

### 

#### 

##### RNAi Knockdown of ATGYRA Mimics Ciprofloxacin Treatment

We previously showed that *A. thaliana* containing a T-DNA insertion in *ATGYRA* had a similar phenotype to plants germinated on ciprofloxacin and that plants germinated on drug-free medium and transferred to medium containing ciprofloxacin showed chlorosis in the developing leaf tissues (7). To test this further, we developed an inducible RNAi line in which *ATGYRA* is coupled to the glucocorticoid receptor protein. Upon induction with dexamethasone, the *ATGYRA* RNAi line had a similar phenotype to plant transferred to a plate containing ciprofloxacin (Fig. 1). This result supports the idea that ciprofloxacin acts on plant gyrase.

##### An EMS Screen for Ciprofloxacin Resistance Identifies an ATGYRA Substitution Allele

**FIGURE 2.**
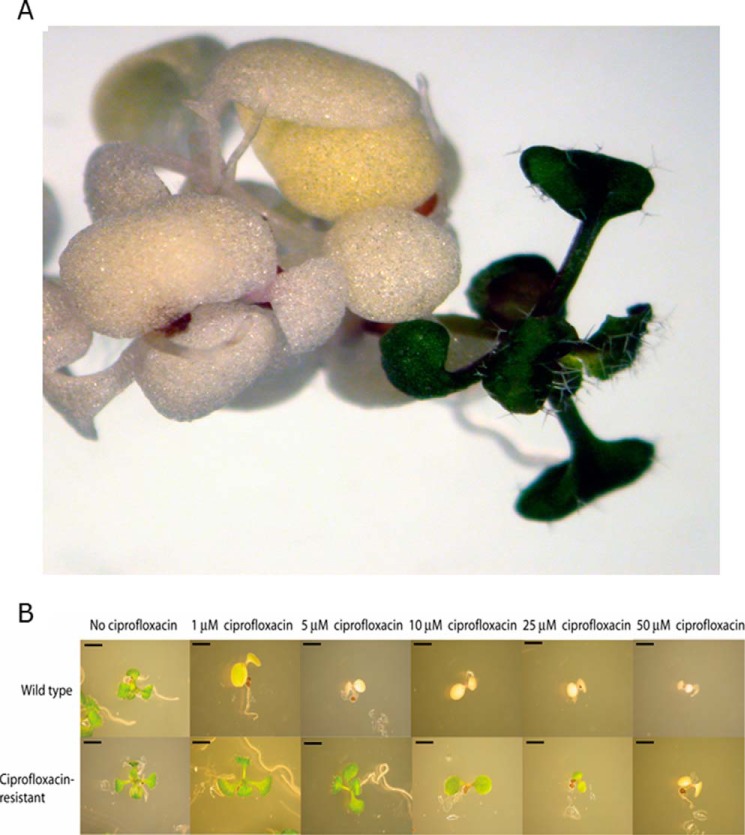
**Mutant plants showing resistance to ciprofloxacin.** *A*, 4-week-old *A. thaliana* plants grown on GM media containing 5 μm ciprofloxacin. On the *left*,several plants susceptible to ciprofloxacin can be seen. With these plants, only the cotyledons have grown, and these are chlorotic. The resistant *gyra-3* plant is on the *right. B*, wild-type *Arabidopsis* and the M_3_ offspring of the ciprofloxacin-resistant plant (*gyra-3*) grown on increasing concentrations of ciprofloxacin. The mutant plants are less susceptible to ciprofloxacin than the wild-type plants and are able to grow normally on 5 μm ciprofloxacin. Each *scale bar* is 2 mm.

To confirm that the target of ciprofloxacin in *A. thaliana* is gyrase, we used a mutagenesis strategy. Approximately 400,000 EMS-treated seeds were grown on medium containing ciprofloxacin (5 μm); one plant was found to survive (Fig. 2*A*). This individual developed and flowered normally, although it was not quite as vigorous as a wild-type plant. The ciprofloxacin resistance was genetically heritable, and plants had no obvious phenotypes in the absence of ciprofloxacin. As the main target of ciprofloxacin in bacteria is known to be DNA gyrase and resistance mutants frequently map to bacterial *gyrA*, we sequenced *ATGYRA* in the resistant plant. This revealed a cytosine to thymine transition (GCT to GTT) resulting in a change from alanine to valine at position 212 in the AtGyrA protein. Alignment of AtGyrA and *E. coli* GyrA revealed that the mutation corresponds to an Ala-119 to Val mutation in *E. coli* GyrA. The first two (lethal) alleles of *gyrA* are T-DNA alleles (7), so this allele was named *gyra-3.*

The A119V mutation has not previously been found in quinolone-resistant *E. coli*, but it has been shown to confer ciprofloxacin resistance in both *Mycoplasma hominis* and *Salmonella typhimurium* (25, 26). Examination of the structure of the N-terminal domain of *E. coli* GyrA (27) reveals that Ala-119 lies close to Tyr-122, the active-site tyrosine, which forms a covalent bond with DNA during the DNA cleavage reaction. Although Ala-119 is not in the canonical quinolone resistance-determining region (28), where most mutations conferring resistance to quinolones map, it is nearby. Other quinolone resistance mutations have been found outside the quinolone resistance-determining region; for example, an Ala-51 to Val mutation in *E. coli* GyrA results in a 6-fold increase in ciprofloxacin resistance (29).

We generated the A119V mutation in the *E. coli gyrA* gene and expressed the mutant protein to determine whether this mutation could confer quinolone resistance. We found that, when complexed with GyrB, the GyrA(A119V) protein showed supercoiling activity; this was found to have the same susceptibility to ciprofloxacin as the wild-type enzyme (data not shown). This is perhaps not surprising as this mutation has never been reported in quinolone-resistant *E. coli* isolates. Therefore we assume that this GyrA mutation only confers quinolone resistance in certain organisms, *e.g. M. hominis*, *S. typhimurium*, and possibly *A. thaliana.*

Mutations corresponding to A119V in both *M. hominis* and *S. typhimurium* result in an approximate 4-fold increase in ciprofloxacin resistance (25, 26). Resistant offspring from the original ciprofloxacin-resistant plant (M_3_ generation) were grown on various concentrations of ciprofloxacin to determine their degree of resistance, by comparison with their wild-type counterparts. The M_3_ plants showed a greater tolerance of ciprofloxacin than wild-type plants, being able to grow normally on 5 μm ciprofloxacin, whereas wild-type plants were barely able to grow on 1 μm ciprofloxacin (Fig. 2*B*). Resistant plants were still able to flower on 10 μm ciprofloxacin, and when grown on 25 μm ciprofloxacin, they were similar in appearance to wild-type plants grown on 1 μm ciprofloxacin. Therefore the M_3_ plants have a roughly 25-fold increase in resistance.

##### Ciprofloxacin-resistant Plants have Normal Chloroplast Morphology in the Presence of Drug

**FIGURE 3.**
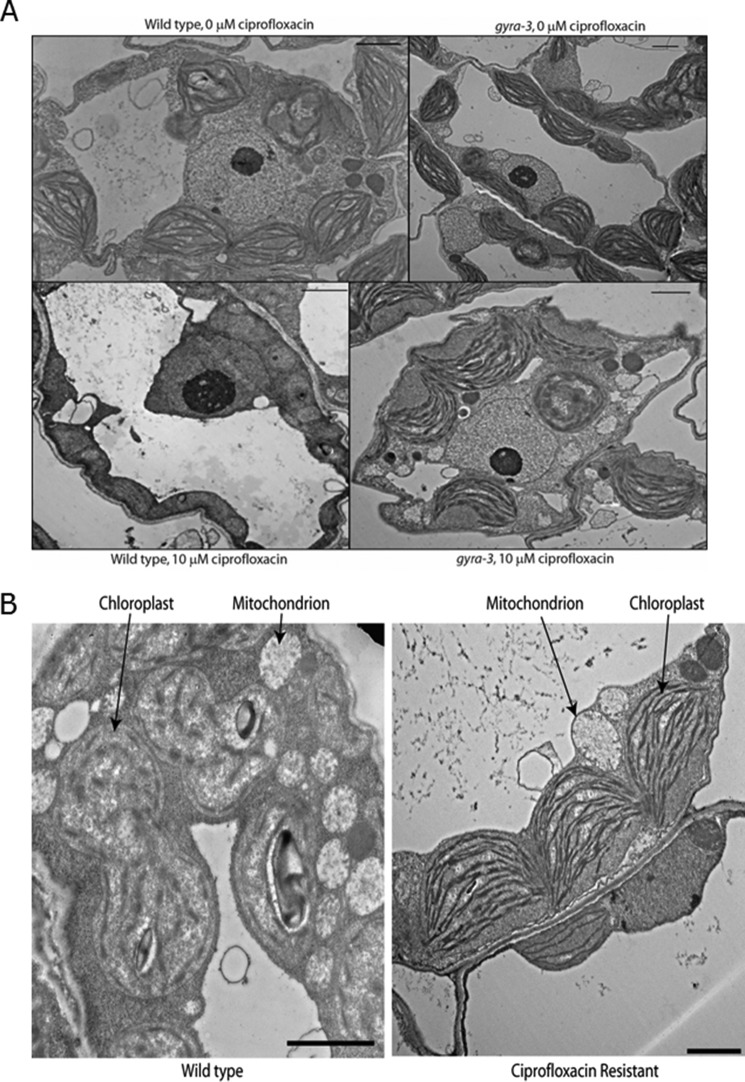
**Transmission electron micrographs of *A. thaliana* cells.** *A*, *top left*, wild type, no ciprofloxacin. *Top right*, ciprofloxacin-resistant *gyra-3* plant, no ciprofloxacin. *Bottom left*, wild type, 10 μm ciprofloxacin (this micrograph is from a chlorotic area of the leaf). *Bottom right*, ciprofloxacin-resistant *gyra-3* plant, 10 μm ciprofloxacin. All *scale bars* are 2 μm. *B*, transmission electron micrographs of ciprofloxacin-resistant and wild-type plants after growth on media containing 10 μm ciprofloxacin, with the endosymbiotic organelles labeled. The mitochondria have similar morphologies in both plants. In the ciprofloxacin-resistant plant, the chloroplasts have the same appearance as in wild-type plants grown on drug-free media. However, in the wild-type plant grown on ciprofloxacin, the thylakoids are less well ordered, and chloroplast division may have been inhibited. Both *scale bars* are 2 μm.

*A. thaliana* DNA gyrase is present in mitochondria and chloroplasts (6, 7). Therefore if the target of ciprofloxacin in *A. thaliana* is DNA gyrase, ciprofloxacin should have a visible effect on organellar morphology. The chlorotic appearance of plants treated with ciprofloxacin suggests that chloroplast function and/or development are affected (6, 7); indeed evidence of altered plastid morphology in the presence of ciprofloxacin has been found previously (7). Wild-type and resistant plants were grown on agar plates for 4 weeks and then transferred to plates containing 0, 1, or 10 μm ciprofloxacin for 2 weeks. Approximately 100 different fields of view were examined by transmission electron microscopy. This revealed that both wild-type and ciprofloxacin-resistant plants have normal organellar morphology when grown on drug-free medium (Fig. 3). In the presence of ciprofloxacin, there is a reduction in the numbers of both chloroplasts and mitochondria in wild-type plants, and they show disrupted chloroplast morphology, with chloroplasts that are more rounded in shape with altered thylakoid arrangements (Fig. 3). More chloroplasts were seen in the process of dividing, rather than as discrete organelles, implying that chloroplast division may be impaired by the addition of ciprofloxacin. However, the mitochondria appear normal (Fig. 3), so it is possible that gyrase may not be essential in these organelles. In contrast, in the presence of ciprofloxacin, drug-resistant plants show normal morphology (Fig. 3), *i.e.* the chloroplasts have the same appearance as those of the wild-type plants in drug-free media.

##### Genetic Rescue of gyra-3 with a Wild-type GYRA Shows That the Ciprofloxacin-resistant GYRA Allele in A. thaliana Is Recessive

**FIGURE 4.**
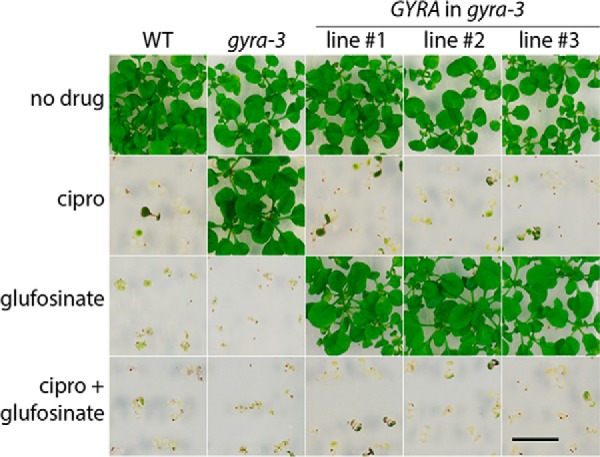
**The effect of the *ATGYRA* transgene on *gyra-3* resistance to ciprofloxacin.** Seeds were sown on agar plates without any addition (*no drug*), 5 μm ciprofloxacin (*cipro*), 25 μm glufosinate ammonium (*glufosinate*), or 5 μm ciprofloxacin plus 25 μm glufosinate ammonium. Wild type is susceptible to ciprofloxacin (*cipro* + *glufosinate*), whereas *gyra-3* is resistant. The transgene contains the *bar* gene selectable marker conferring resistance to glufosinate ammonium. The *ATGYRA* transgene is included on a T-DNA along with the *bar* gene selectable marker conferring resistance to glufosinate ammonium. The WT *GYRA* in *gyra-3* transgenic lines confers susceptibility to ciprofloxacin, indicating that the *gyra-3* lesion is responsible for ciprofloxacin resistance. Images were taken after 21 days of growth.

An alternative approach to demonstrate that the *gyra-3* lesion is causing ciprofloxacin resistance is to genetically rescue the *gyra-3* allele to exhibit a wild-type ciprofloxacin-susceptible phenotype. Ciprofloxacin and other quinolone drugs are known to bind bacterial DNA gyrase and cause it to introduce double-stranded breaks into DNA (30, 31); this is thought to be a lethal event *in vivo* (32). This mechanism of cell killing results in dominance of a quinolone-sensitive *gyrA* over a resistant allele (33). Therefore introduction of a wild-type *gyrA* gene into a bacterial cell that is quinolone-resistant, due to the presence of a quinolone-resistant *gyrA* gene in the chromosome, will cause the cell to become quinolone-sensitive. We reasoned that if the *gyra-3* allele is causing ciprofloxacin resistance, introduction of wild-type *ATGYRA* should restore the ciprofloxacin-sensitive phenotype. The method for introducing the wild-type *ATGYRA* gene into the *gyra-3* background is described under “Experimental Procedures,” and the results are shown in Fig. 4 and summarized in Table 1. We transformed *gyra-3* with an 8-kb wild-type genomic copy of *ATGYRA* and compared the phenotype of three independent transgenic lines to wild type and *gyra-3*. All three *ATGYRA* in *gyra-3* transgenic lines were as ciprofloxacin-sensitive as wild type, and by analyzing homozygous T3, we could show that resistance was lost upon addition of the transgene (Table 1). These results show that, as in bacteria, the quinolone-sensitive *ATGYRA* allele is dominant to the resistant allele. As the only lesion in the *ATGYRA* gene, this rescue with WT *ATGYRA* confirms that the A212V mutation in *A. thaliana ATGYRA* confers resistance to ciprofloxacin.

**TABLE 1:** **The effect of the *GYRA* transgene on the *gyra-3* resistance to ciprofloxacin measured by survival** Seeds were sown on agar plates without any addition (no drug), 5 μm ciprofloxacin (cipro), 25 μm glufosinate ammonium (gluf.) or 5 μm ciprofloxacin/25 μm glufosinate ammonium (cipro/gluf.). Wild type is susceptible to ciprofloxacin, whereas *gyra-3* is resistant. The transgene contains the *bar* gene selectable marker conferring resistance to glufosinate ammonium. The WT GYRA in *gyra-3* transgenic lines confers susceptibility to ciprofloxacin, indicating that the *gyra-3* lesion is responsible for ciprofloxacin resistance. Plants with at least two true leaves were classified as to have survived. Values indicate surviving plants *versus* total plants.

	No drug	Gluf.	Cipro	Cipro/Gluf.
WT	33/33	0/24	0/26	0/24
*gyra-3*	12/12	0/17	15/15	0/20
GYRA in *gyra-3* line 1	24/24	22/22	0/27	0/27
GYRA in *gyra-3* line 2	24/24	18/18	0/19	0/25
GYRA in *gyra-3* line 3	25/25	17/17	0/17	0/17

##### Reconstitution of A. thaliana Gyrase Supercoiling Activity in Vitro

**FIGURE 5.**
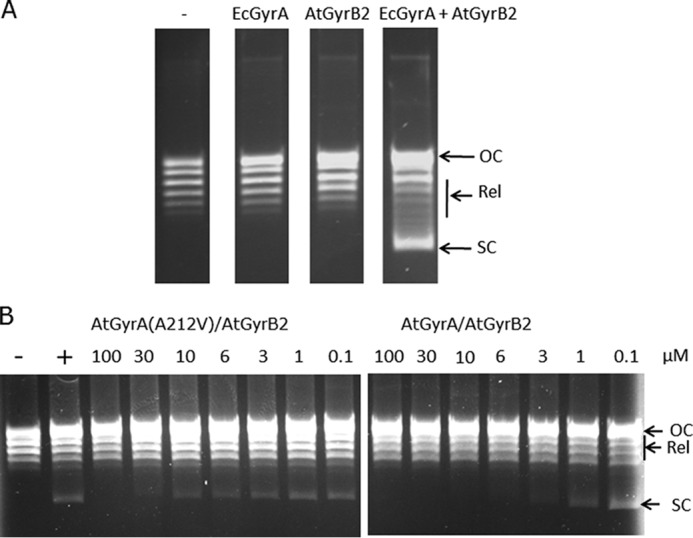
**Supercoiling activity of *A. thaliana* DNA gyrase.** *A*, *ATGYRB2* was heterologously expressed in *E. coli* and assayed for supercoiling activity (as described under “Experimental Procedures”) in the absence and presence of GyrA from *E. coli*. − indicates no protein; EcGyrA indicates GyrA from *E. coli*; and AtGyrB2 indicates GyrB2 from *A. thaliana. OC* = open-circular (nicked) DNA; *Rel* = relaxed topoisomers; *SC* = supercoiled DNA. *B*, DNA supercoiling assay with wild-type and mutant *A. thaliana* gyrase, containing AtGyrA with the A212V mutation, in the absence and presence of ciprofloxacin. − indicates no enzyme; + indicates enzyme but no drug. Ciprofloxacin concentrations, in μm, are as indicated. Conditions were adjusted to give partial supercoiling so that the differences between the wild-type and quinolone-resistant enzyme could be easily quantified.

In previous work, it was reported that *A. thaliana* gyrase proteins expressed in *E. coli* did not show supercoiling activity (7). One difficulty with these experiments is the presence of organellar targeting peptides at the start of the AtGyrA and AtGyrB proteins and uncertainties over the exact translational start of the coding sequences. Another problem is the need to have both the A and the B subunits folded and active to obtain enzyme activity. However, subsequently, we have been able to express AtGyrB2 in *E. coli* and show that, when it is combined with *E. coli* GyrA, supercoiling activity can be seen (Fig. 5*A*).

Another way to demonstrate activity associated with the *A. thaliana* gyrase proteins is their ability to complement *E. coli* temperature-sensitive (ts) mutants. In earlier work (7), it appeared that all four *A. thaliana* gyrase genes (*ATGYRA*, *ATGYRB1*, *ATGYRB2*, and *ATGYRB3*) were able to complement *E. coli* ts strains (KNK453 in the case of *ATGYRA* and N4177 in the case of the *ATGYRB* genes); subsequent work (9) has shown this to be incorrect. Although *ATGYRB2* has been shown to complement *E. coli* N4177, *ATGYRB3* cannot (9); indeed it appears that *ATGYRB3* encodes a protein that is not a gyrase subunit and is likely to have an unrelated function. We have now shown that *ATGYRA* is also unable to complement an *E. coli gyrA^ts^* strain (KNK453) using a variety of constructs (data not shown). Further we have shown that *ATGYRB1* is unable to complement *E. coli* N4177, and indeed its expression appears lethal to *E. coli* (data not shown); this is despite the fact that in yeast two-hybrid experiments, AtGyrB1 was shown to interact with AtGyrA, whereas AtGyrB2 and AtGyrB3 did not (9). From this work, it appears that there are significant difficulties in expressing *A. thaliana* gyrase proteins in *E. coli* and detecting activity *in vivo* or *in vitro*. For comparison, it has been shown that the gyrase genes from *N. benthamiana*, *NbGyrA* and *NbGyrB*, are able to complement the corresponding *E. coli* ts strains (6).

To address these problems, we have attempted expression of the *A. thaliana* gyrase proteins heterologously in eukaryotic systems. Working with the OPPF, we were able to successfully express AtGyrA and AtGyrB2 proteins in a baculovirus system using co-expression of the *ATGYRA* and *ATGYRB2* genes from two plasmid vectors. Partial purification of the *A. thaliana* gyrase proteins showed supercoiling activity that was ATP-dependent and could be inhibited by gyrase-specific antibiotics (Fig. 5*B*) and showed quinolone-induced DNA cleavage (data not shown). Moreover when the *ATGYRA* gene was replaced by the mutant gene described above encoding the ciprofloxacin-resistant AtGyrA subunit (containing the A212V mutation), the partially purified enzyme showed ciprofloxacin-resistant supercoiling activity (∼10-fold increase as compared with wild type; Fig. 5*B*) but normal sensitivity to novobiocin (data not shown).

These experiments show that the complex between AtGyrA and AtGyrB2 generates an active supercoiling enzyme that shares similar properties to its bacterial counterpart, including sensitivity to gyrase-specific antibiotics. Moreover they show that the A212V mutation in AtGyrA is responsible for the quinolone resistance phenotype of the *A. thaliana* mutant (*gyra-3*) described above and that an active DNA gyrase is encoded by *A. thaliana* and is the target of the antibiotic ciprofloxacin.

## Discussion

DNA gyrase has been known for some time to exist in plants (6, 7), but the lethality of knockouts and difficulties in producing active recombinant protein have meant that plant gyrase has remained largely uncharacterized. It was previously shown that one of the *GYRB* alleles (*ATGYRB3*) is not a gyrase gene and is likely to have an unrelated function (9). It has been assumed that the remaining gyrase genes, *ATGYRA*, *ATGYRB1*, and *ATGYRB2*, encode subunits for active gyrase enzymes that have a role in organellar DNA replication. However, this had not been established. Further it has been shown that *A. thaliana* is sensitive to gyrase-specific antibiotics (*e.g.* ciprofloxacin and novobiocin (7)), but it has not been proven whether the plant gyrase is the target.

The most direct way to ascertain the target of a drug is to isolate mutants that map to the gene encoding the target enzyme. We used EMS-mutagenized *A. thaliana* seeds to screen for plants with resistance to ciprofloxacin. From ∼400,000 seeds, we found one ciprofloxacin-resistant plant showing about a 25-fold decrease in susceptibility to the drug. Sequencing showed the mutation to lie in the *ATGYRA* gene leading to an Ala to Val substitution at position 212 in the AtGyrA protein; this corresponds to Ala-119 to Val in *E. coli* GyrA. This mutation has not been reported in *E. coli*, and generation of the mutation in the *E. coli* GyrA protein yielded an active protein with wild-type susceptibility to ciprofloxacin. However, it is known that the same mutation leads to ciprofloxacin resistance in other species, *e.g. M. hominis* and *S. typhimurium* (25, 26).

Treatment of *A. thaliana* with ciprofloxacin affects chloroplast numbers and morphology; the ciprofloxacin-resistant plant (*gyra-3*) showed normal chloroplast morphologies in the presence of the drug. With wild-type plants, many of the chloroplasts appeared to be in the process of dividing, implying that the drug was interrupting organellar replication, as might be predicted. Interestingly, we found little difference in mitochondrial morphology for wild-type plants in the presence of ciprofloxacin, suggesting that gyrase may not play such a crucial role in these organelles. These results further endorse the view that gyrase is targeted to chloroplasts and is targeted by ciprofloxacin (6, 7).

We applied two further tests to prove that DNA gyrase is active in *A. thaliana* and that it is the target of ciprofloxacin. Firstly, we used genetic rescue to show that the ciprofloxacin resistance *ATGYRA* mutation in *gyra-3*, when in the presence of a wild-type (ciprofloxacin-sensitive) *ATGYRA* allele, is recessive, *i.e.* the phenotype of the resulting merodiploid plant was ciprofloxacin-sensitive; this result mirrors that obtained in bacteria (33). Secondly, using a baculovirus expression system, we were able to express the *ATGYRA* and *ATGYRB2* genes and obtain active *A. thaliana* gyrase that showed sensitivity to ciprofloxacin. Moreover when we expressed the AtGyrA(A212V) mutant protein, we found that it conferred drug resistance. Taken together these experiments confirm that DNA gyrase is active in *A. thaliana* and that it is the target of the quinolone drug ciprofloxacin.

The difficulties in heterologously expressing the *A. thaliana* gyrase proteins have hampered progress in characterizing this enzyme and exploring its role in organellar replication, as well as plant growth and development. It is hoped that the results described in this study, particularly the successful heterologous expression in insect cells, will pave the way for future work on this enzyme. In particular, it would be useful to ascertain whether AtGyrB1 and AtGyrB2 have distinct roles and whether both are able to complex with AtGyrA to constitute active supercoiling enzymes. The confirmation that *A. thaliana* gyrase is indeed the target for quinolone antibacterials raises the possibility of developing compounds specifically targeted to this enzyme that could be developed as herbicides.

## Author Contributions

A. M. conceived and coordinated the study and wrote the paper with contributions from J. S. M. K. M. E. R., L. A. M., M. K. W., and J. L. carried out the experiments. All authors reviewed the results and approved and edited the final version of the manuscript.

## Footnotes

4 The abbreviations used are:

GM: germination medium
EMS: ethyl methanesulfonate
ts: temperature-sensitive.
